# Supplementary material for: Single-atom level determination of 3-dimensional surface atomic structure via neural network-assisted atomic electron tomography
Source: Nat Commun. 2021 Mar 30;12:1962. doi: 10.1038/s41467-021-22204-1 (PMC8009920; doi:10.1038/s41467-021-22204-1)
Supplement: Supplementary file 1 — Supplementary Information [file 41467_2021_22204_MOESM1_ESM.pdf]

Supplementary Information

for

Single-atom level determination of 3-dimensional surface atomic structure via neural network-assisted atomic electron tomography

Juhyeok Lee[1], Chaehwa Jeong[1] and Yongsoo Yang[1*]

[1]*Department of Physics, Korea Advanced Institute of Science and Technology (KAIST), Daejeon 34141, Korea*

*Email: yongsoo.yang@kaist.ac.kr

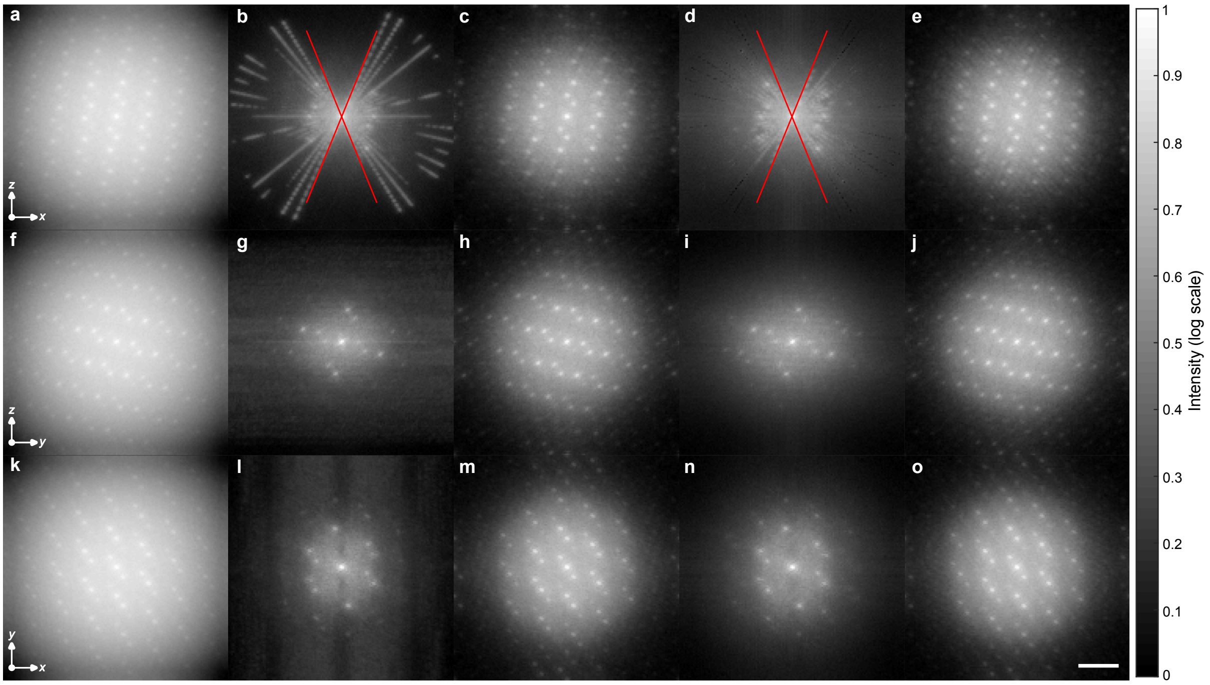

Supplementary Figure 1 | Projected 3D Fourier intensities of simulated tomograms along different axes. a-e Projections of 3D Fourier intensities along the *y*-direction for ground truth 3D volume (**a**), for tomograms reconstructed from linear projections before (**b**), and after (**c**) the DL augmentation, for tomograms reconstructed from PRISM projections before (**d**) and after (**e**) the DL augmentation, respectively. **f-j** Projections similar to (**a-e**) along the *x*-direction. **k-o** Projections similar to (**a-e**) along the *z*-direction. The red lines in (**b**) and (**d**) are guides for eye to visualize the missing wedge. It can be clearly seen that the missing wedge information and high frequency f.c.c. peak information are successfully recovered by applying the DL augmentation. Scale bar, 0.5 Å^{-1}.

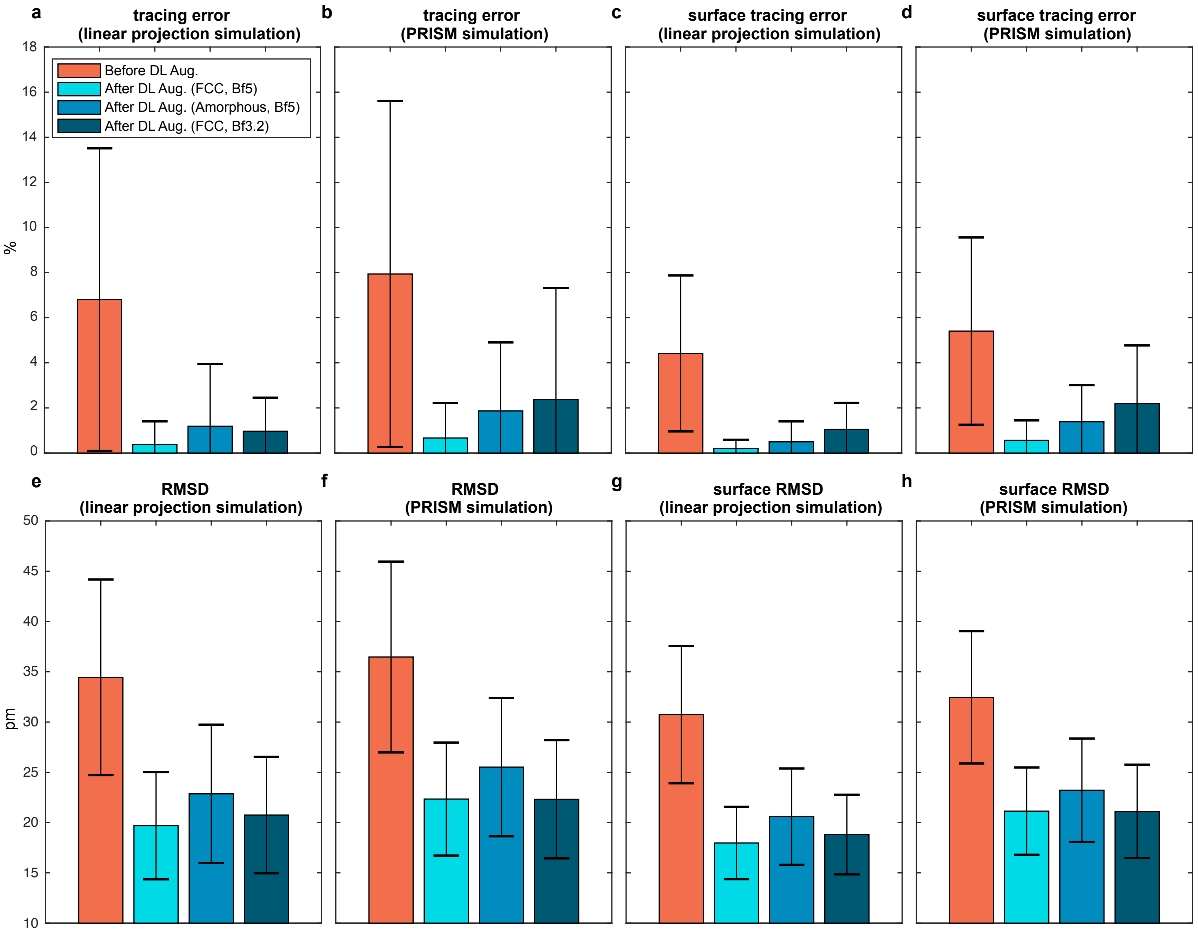

Supplementary Figure 2 | Tracing errors and RMSDs of atomic structures obtained from the simulated Pt nanoparticle tomogram data. The tracing errors (**a-d**) and RMSDs (**e-h**) were calculated by comparing the ground truths and traced atomic models from linear (**a**, **c**, **e**, **g**) and PRISM (**b**, **d**, **f**, **h**) simulations, before and after different DL augmentations. Each bar was obtained by averaging the results from the test dataset of 1,000 tomograms for both linear and PRISM simulations. The error bars represent the standard deviations.

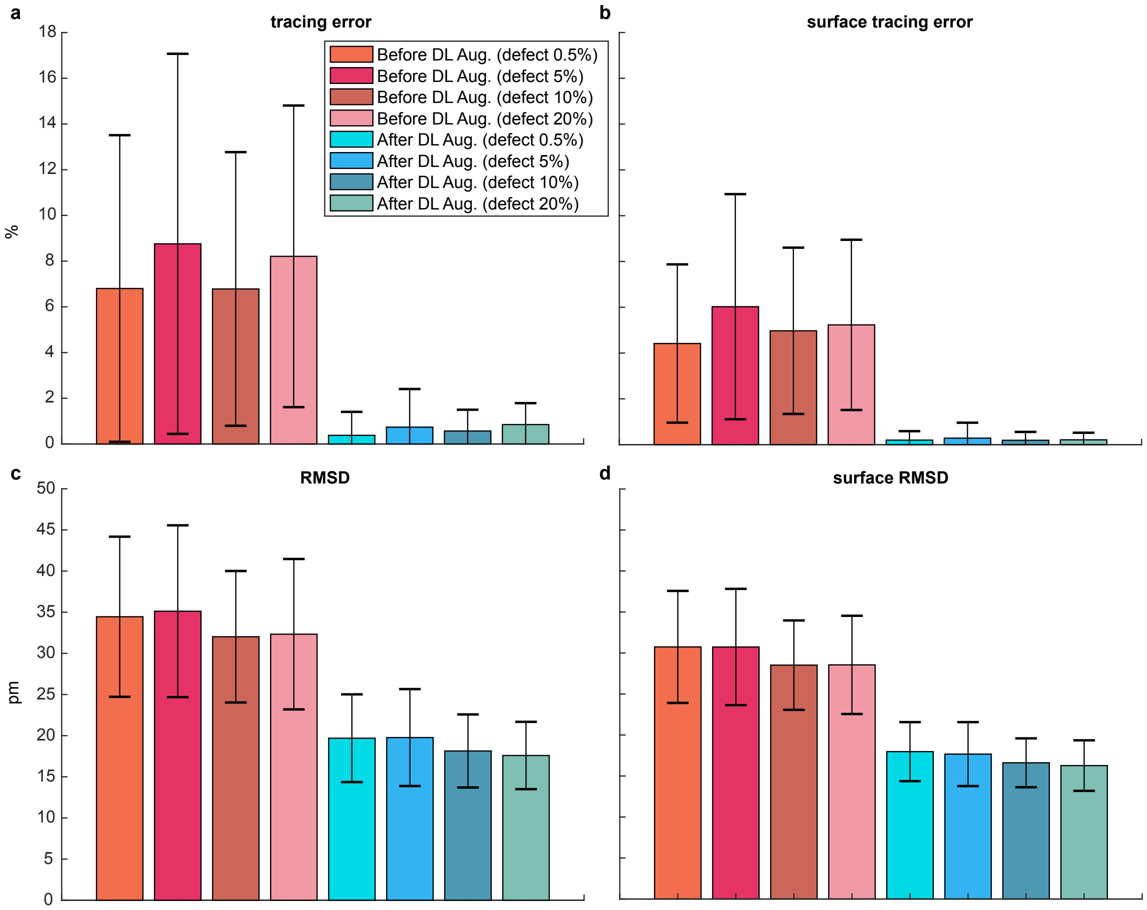

Supplementary Figure 3 | Tracing errors and RMSDs of atomic structures obtained from the Pt nanoparticle simulations with different defect levels. The tracing errors (**a, b**) and RMSDs (**c, d**) were calculated by comparing the ground truths and traced atomic models from the linear projection-based simulations before and after the DL augmentation. Each bar was obtained by averaging the results from the test datasets of 100 tomograms (except for the 0.5% defect level case where we used the test dataset of 1,000 tomograms). The error bars represent the standard deviations. The DL augmentation neural network was trained by the training dataset based on f.c.c. atomic models with defect level 0.5% and Bfactor 5 Å^2.

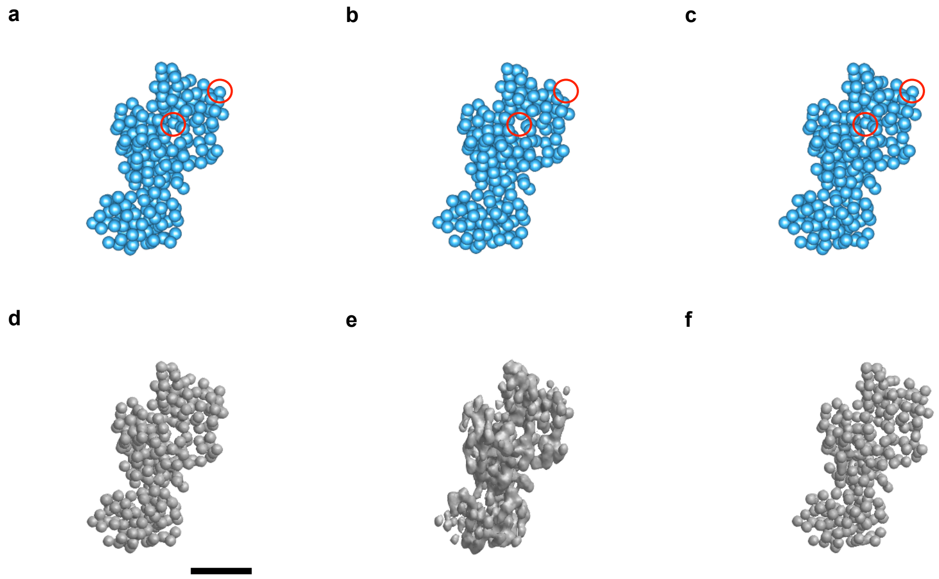

Supplementary Figure 4 | Traced atomic models and iso-surface renderings from tomography simulations of 1 nm diameter amorphous nanoparticle, before and after the DL augmentation. Traced atomic positions from the 3D volumes of the ground truth (**a**), the simulated 3D tomographic reconstructions before (**b**) and after the DL augmentation (**c**). The 3D iso-surface of the ground truth (**d**), the simulated 3D tomographic reconstructions before (**e**) and after the DL augmentation (**f**). The 3D iso-surfaces were plotted with 10% (**d**, **f**) and 30% (**e**) intensity thresholds from the maximum intensity. The red circles highlight the locations of misidentified atoms in the raw reconstruction, which can be clearly resolved after the DL augmentation. The DL augmentation neural network was trained by training dataset based on f.c.c. atomic models with Bfactor 5 Å^2. Scale bar, 1 nm.

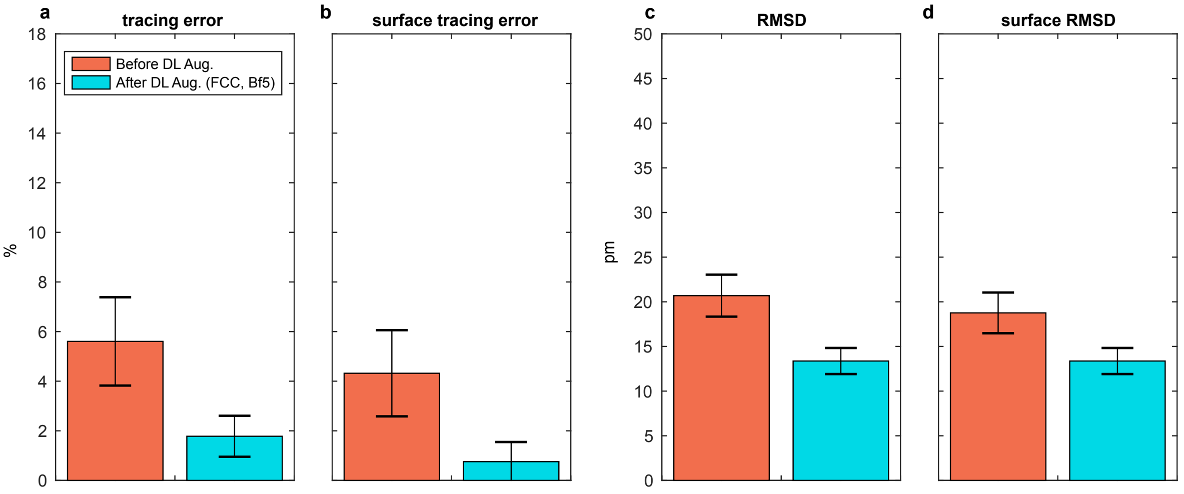

Supplementary Figure 5 | Tracing errors and RMSDs of the atomic structures obtained from the simulated reconstructions of 1 nm diameter amorphous nanoparticles. The tracing errors (**a, b**) and RMSDs (**c, d**) were calculated by comparing the ground truths and traced atomic models from the linear projection-based tomographic simulations before and after the DL augmentation. Each bar was obtained by averaging the results from the test dataset of 100 tomograms. The DL augmentation neural network was trained by the training dataset based on f.c.c. atomic models with Bfactor 5 Å^2. The error bars represent the standard deviations.

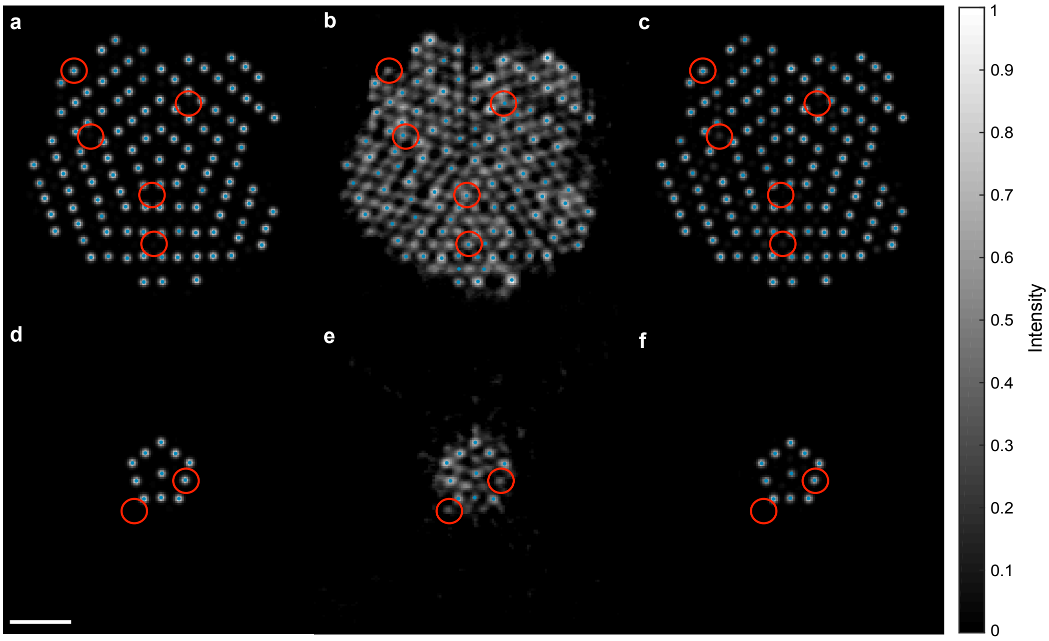

Supplementary Figure 6 | Effect of the DL augmentation in the atomic layer slices of a simulated 3D tomogram of a decahedral nanoparticle with twin boundaries. a-f 2-Å thick slices perpendicular to [001] direction, obtained from the 3D tomogram of the nanoparticle near the center region (**a-c**) and near the surface (**d-f**). The slices are obtained from the ground truth (**a, d**), raw tomogram (**b, e**) and tomogram after applying the DL augmentation (**c, f**). Red circles highlight the locations of misidentified atoms in the raw reconstruction, which can be clearly resolved after the DL augmentation. Grayscale background represents the reconstructed intensity, and blue dots represent the positions of traced Pt atoms. The DL augmentation neural network was trained by the training dataset based on f.c.c. atomic models with Bfactor 5 Å^2. Scale bar, 1 nm.

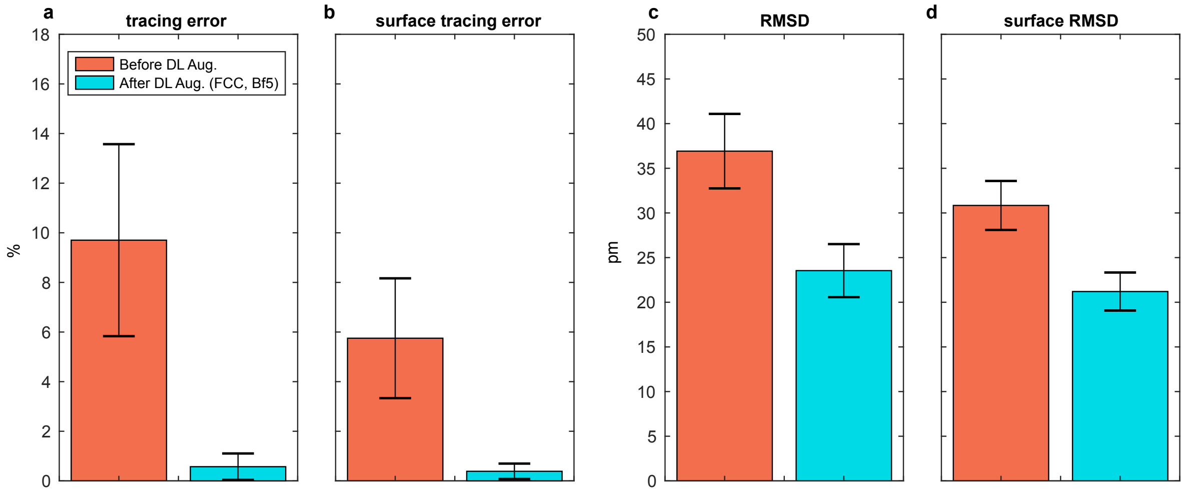

Supplementary Figure 7 | Tracing errors and RMSDs of the atomic structures obtained from the simulated reconstructions of decahedral nanoparticles with twin boundaries. The tracing errors (**a, b**) and RMSDs (**c, d**) were calculated by comparing the ground truths and traced atomic models from linear projection-based tomographic simulations before and after the DL augmentation. Each bar was obtained by averaging the results from the test dataset of 100 tomograms. The DL augmentation neural network was trained by the training dataset based on f.c.c. atomic models with Bfactor 5 Å^2. The error bars represent the standard deviations.

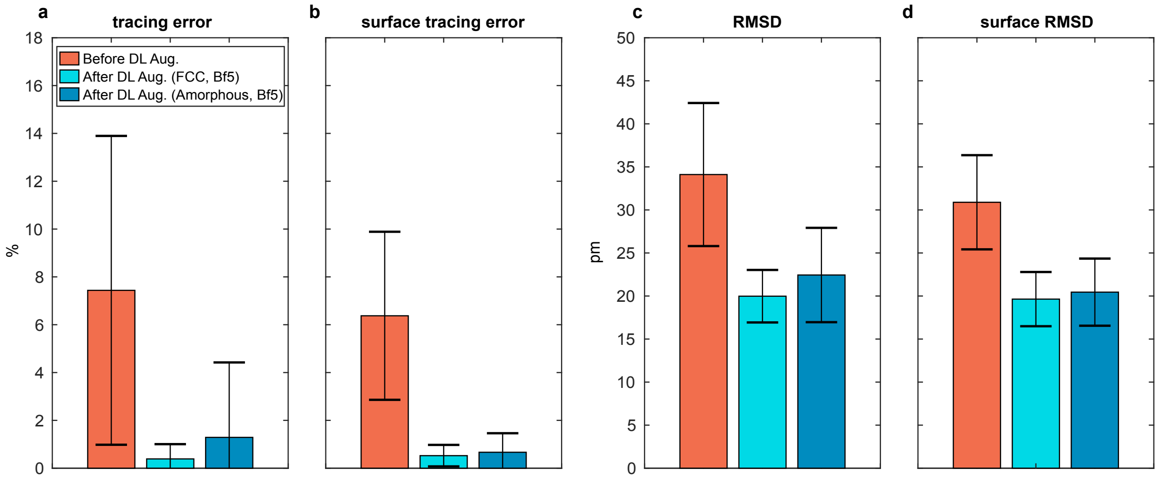

Supplementary Figure 8 | Tracing errors and RMSDs of the tomography simulations of f.c.c.-based atomic structures with spatial displacement of 22 pm for core atoms and 50 pm for surface atoms. The tracing errors (**a, b**) and RMSDs (**c, d**) were calculated by comparing the ground truths and traced atomic models from linear projection-based tomographic simulations before and after the DL augmentation. Two different DL networks, one trained by f.c.c. atomic models (Bfactor 5 Å^2) and another trained by amorphous models, were used for the test. Each bar was obtained by averaging the results from the test dataset of 100 tomograms. The error bars represent the standard deviations.

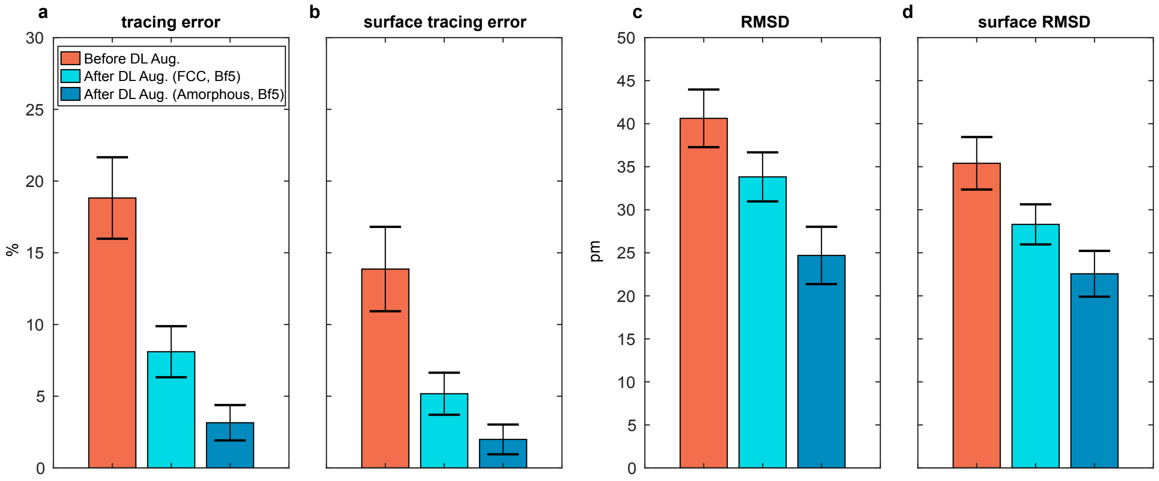

Supplementary Figure 9 | Tracing errors and RMSDs of the tomography simulation results from amorphous atomic structures. The amorphous atomic structures for this test were generated using the same method for obtaining the amorphous training dataset (Methods). The tracing errors (**a, b**) and RMSDs (**c, d**) were calculated by comparing the ground truths and traced atomic models from linear projection-based tomographic simulations before and after the DL augmentation. Two different DL networks, one trained by the f.c.c.-based atomic models (Bfactor 5 Å^2) and another trained by the amorphous models, were used for the test. Each bar was obtained by averaging the results from the test dataset of 100 tomograms. The error bars represent the standard deviations.

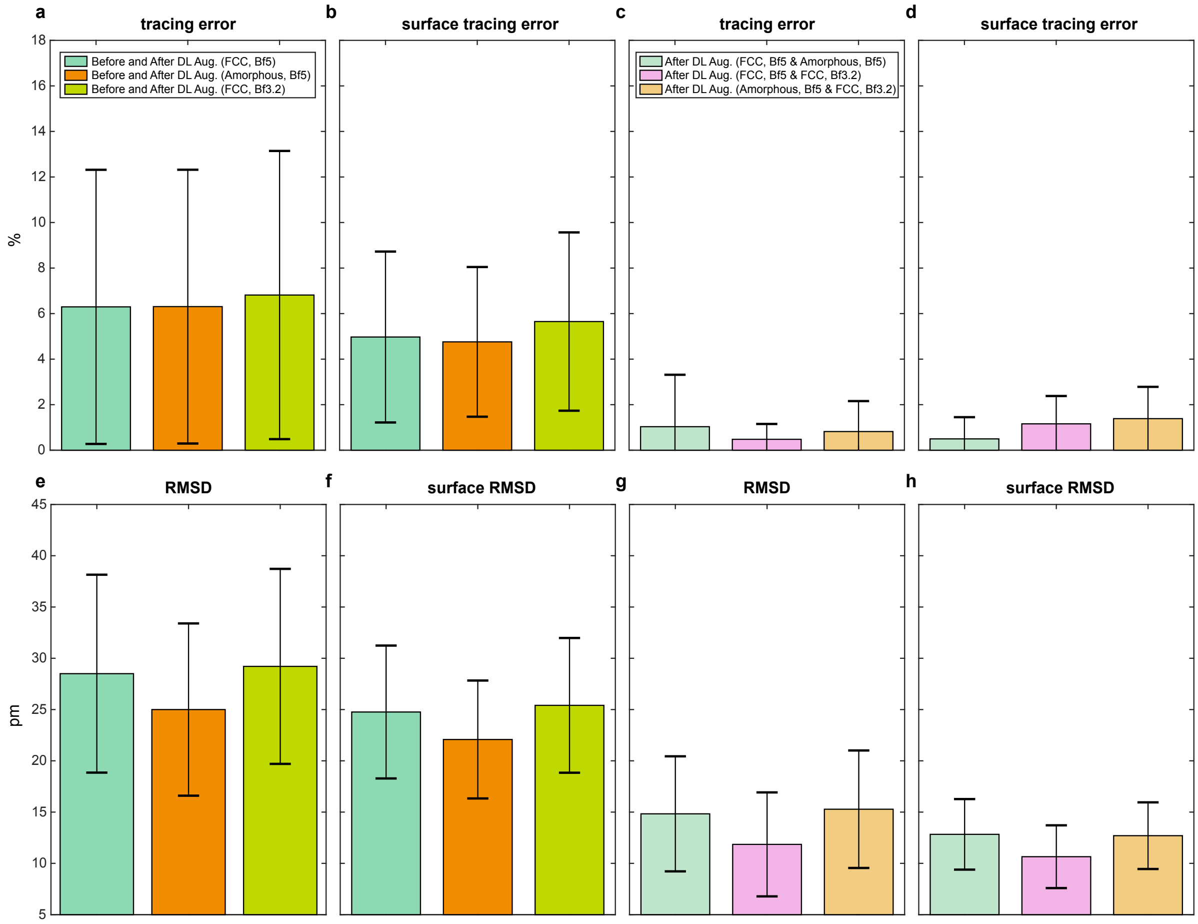

Supplementary Figure 10 | Tracing errors and RMSDs between the atomic structures obtained from simulated Pt nanoparticle tomogram data before and after applying different DL augmentation networks. The tracing errors (**a**-**d**) and RMSDs (**e**-**h**) were calculated from the obtained atomic structures i) by comparing the results from the raw tomograms to those from DL augmented tomograms (**a**, **b**, **e**, **f**), and ii) by comparing between the results from the three different DL augmentations (**c**, **d**, **g**, **h**). (**a**, **c**, **e**, **g**) show the total tracing errors and RMSDs, and (**b**, **d**, **f**, **h**) show the surface tracing errors and RMSDs. Each bar was obtained by averaging the results from the test datasets of 1,000 tomograms. All errors were calculated from the linear projection simulations. The error bars represent the standard deviations.

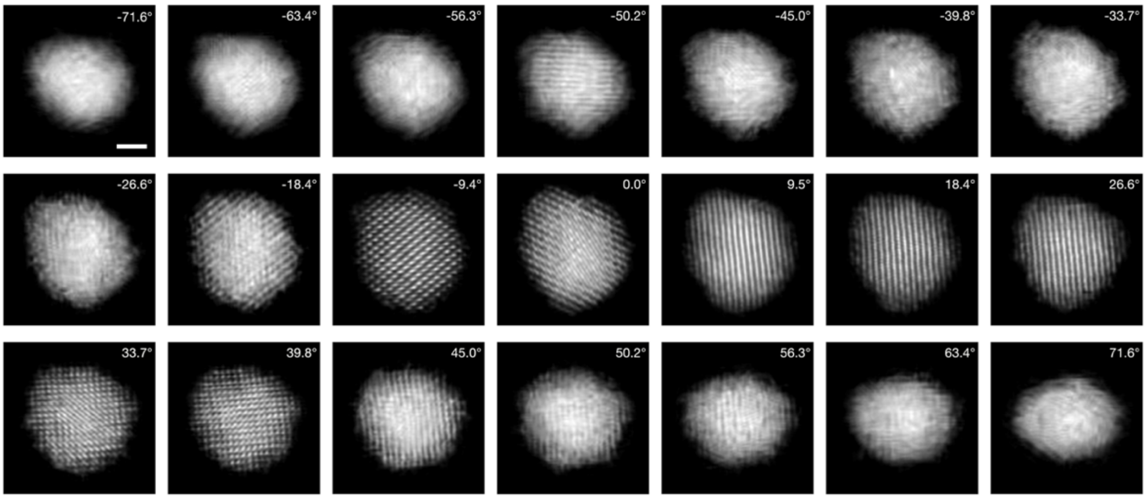

Supplementary Figure 11 | An experimental tomographic tilt series of a Pt nanoparticle. Total 21 tilt series images were acquired from an ADF-STEM experiment and post-processed as described in the method section. Corresponding tilt angle for each projection is denoted at the top right corner of each image. Scale bar, 1 nm.

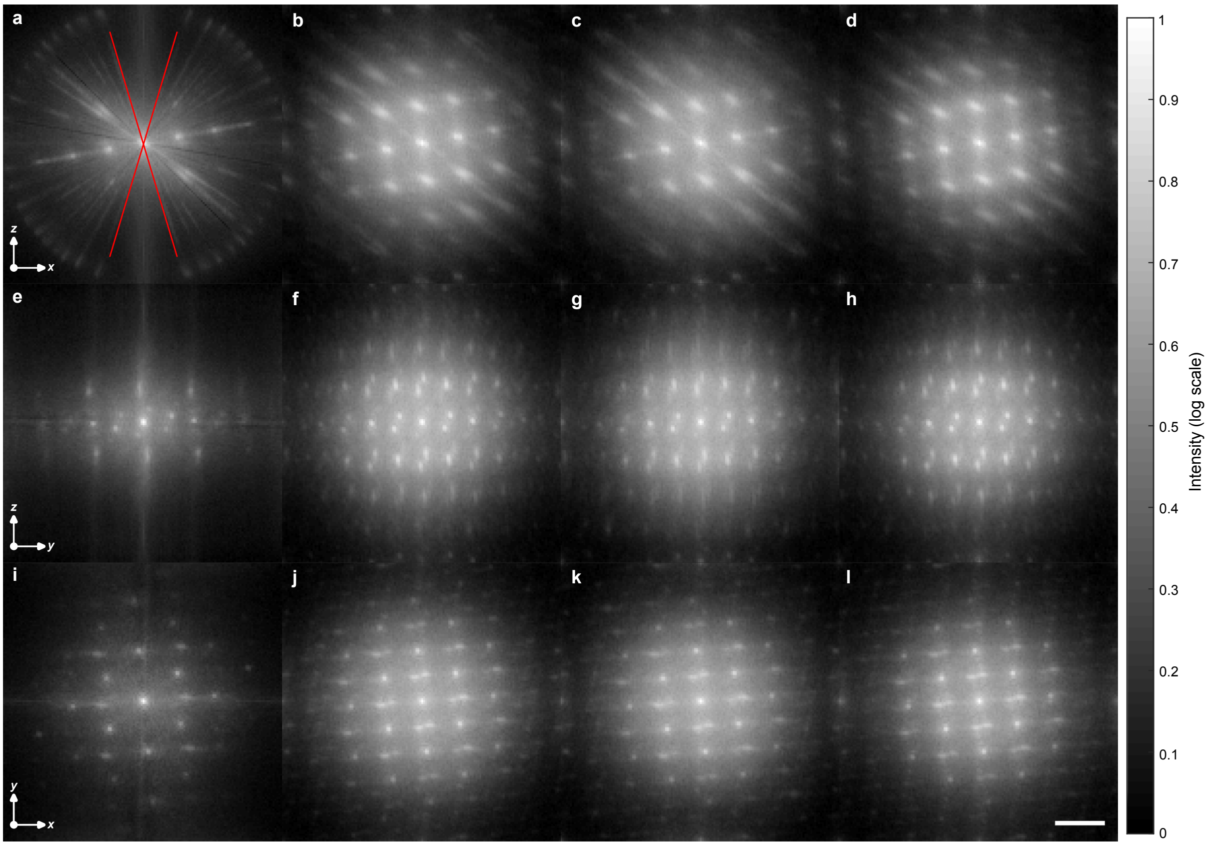

Supplementary Figure 12 | Projected 3D Fourier intensities of experimental tomograms. a-d Projections of 3D Fourier intensities along the *y*-direction for the raw experimental tomogram of the Pt nanoparticle (**a**), for the tomograms obtained by applying the DL augmentations trained by f.c.c. model + Bfactor 5 Å^2 (**b**), amorphous model + Bfactor 5 Å^2 (**c**) and f.c.c. model + Bfactor 3.2 Å^2 (**d**), respectively. **e-h** Projections similar to (**a-d**) along the *x*-direction. **i-l** Projections similar to (**a-d**) along the *z*-direction. The red lines in (**a**) are guides for eye to visualize the missing wedge. It can be clearly seen that the missing wedge information and high frequency f.c.c. peak information are successfully recovered by applying the DL augmentations, even with the DL augmentation trained by the atomic models based on amorphous structure, demonstrating the robustness of this approach. Scale bar, 0.5 Å^{-1}

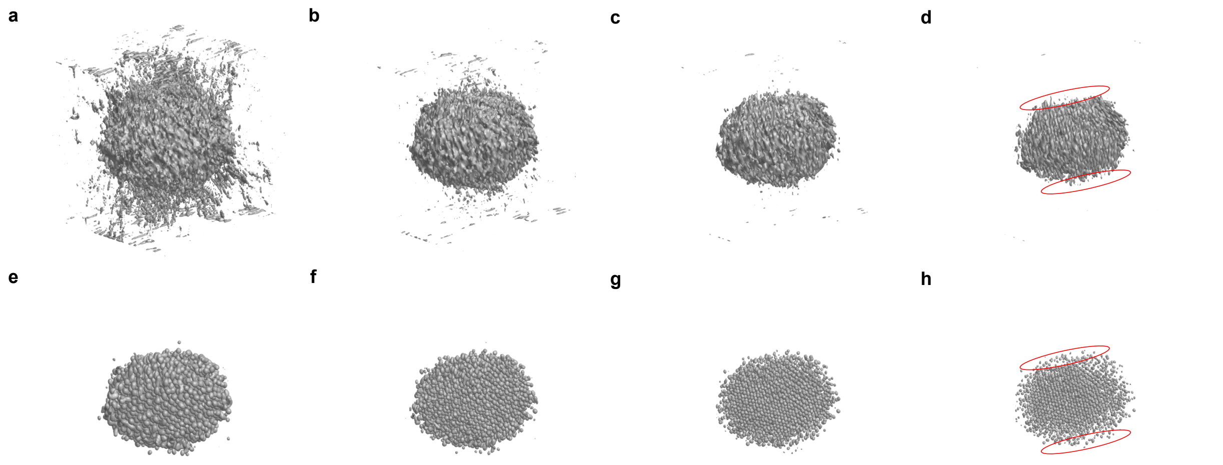

Supplementary Figure 13 | Iso-surface renderings of the 3D tomograms before and after the DL augmentation. 3D iso-surfaces of the experimental raw tomogram (**a**-**d**) and the DL augmented tomogram (**e**-**h**) plotted with 10 % (**a**, **e**), 20 % (**b**, **f**), 30 % (**c**, **g**), and 40 % (**d**, **h**) intensity threshold from the maximum intensity. Due to the missing wedge problem, the surface boundary is not well-defined along the missing wedge direction for the raw tomogram (surface boundary being sensitive to the threshold value), but the tomogram after the DL augmentation shows consistent surface structure throughout different iso-surface thresholds. Red ellipses in (**d**) and (**h**) represent the surface boundary along the missing wedge direction.

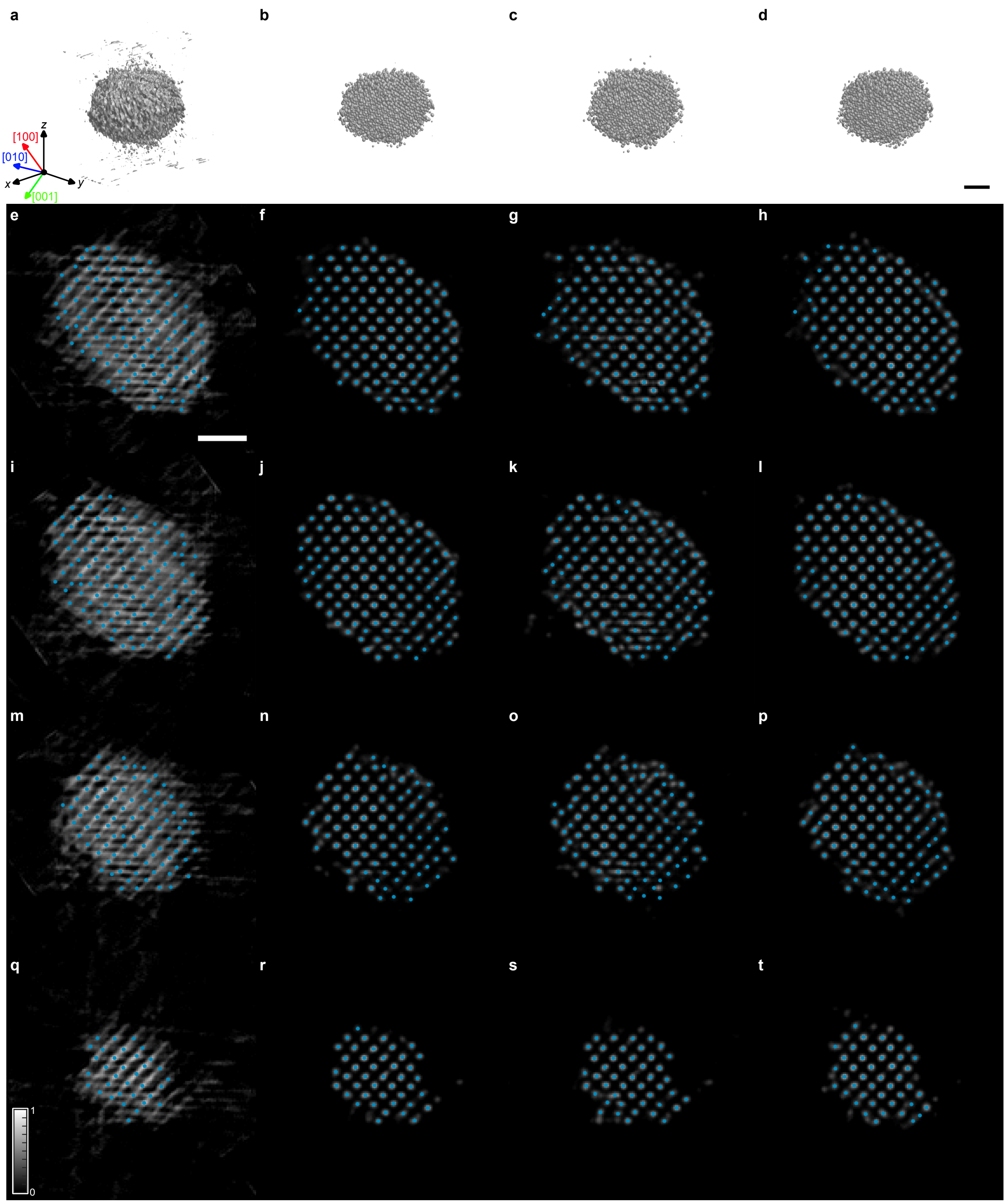

Supplementary Figure 14 | Effect of different DL augmentations for the experimental tomogram. a-d, 3D iso-surfaces plotted with 20% iso-surface threshold from the maximum intensity, representing raw tomogram (**a**), tomograms after applying the DL augmentation trained by f.c.c. models + Bfactor 5 Å^2 (**b**), amorphous models + Bfactor 5 Å^2 (**c**), and f.c.c. models + Bfactor 3.2 Å^2 (**d**). Note that the *z*-direction is the missing wedge direction. **e**-**t**, 2-Å thick slices perpendicular to [001] direction obtained from the 3D tomograms: the raw tomogram (**e, i, m, q**), the tomograms after applying the DL augmentations trained by f.c.c. models + Bfactor 5 Å^2 (**f, j, n, r**), amorphous models + Bfactor 5 Å^2 (**g, k, o, s**), and f.c.c. models + Bfactor 3.2 Å^2 (**h, l, p, t**). Grayscale background represents the reconstructed intensity, and blue dots represent the positions of traced Pt atoms. For fair comparison, the manual corrections of the atom-tracing were not applied here. Scale bars, 1 nm.

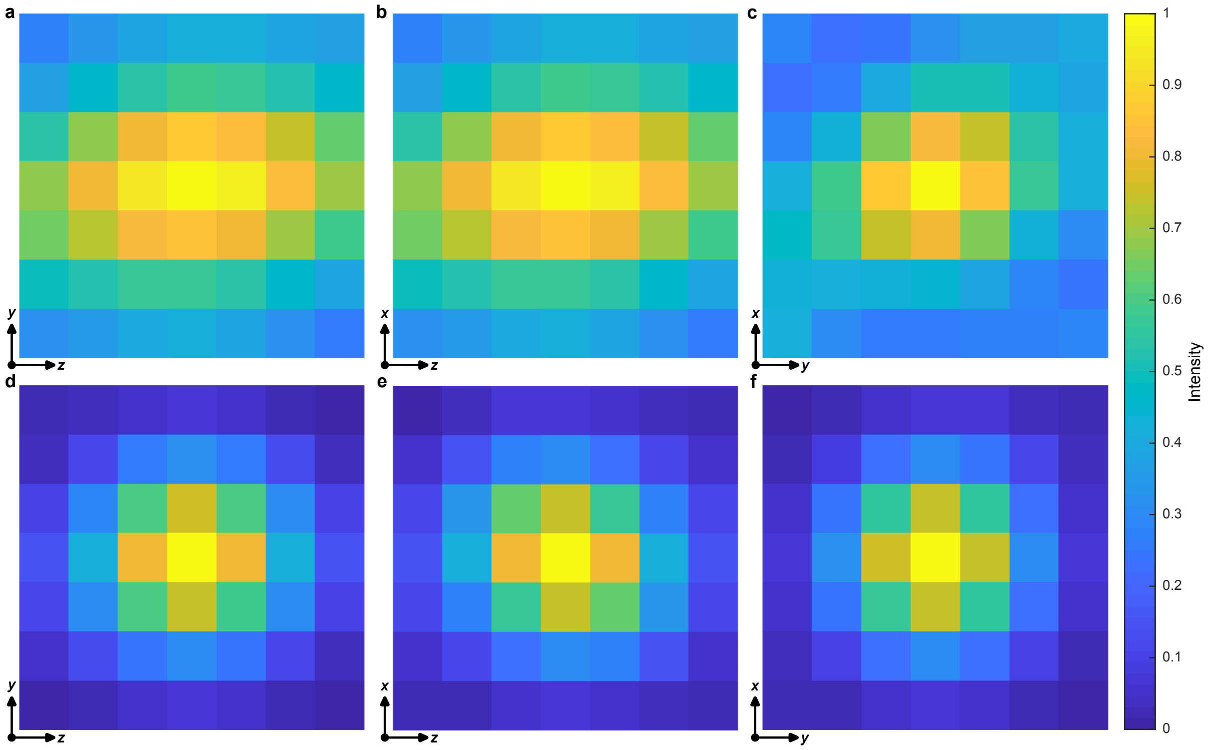

Supplementary Figure 15 | Averaged 3D atom profile obtained from the experimental data before and after the DL augmentation. a-**c** Averaged intensity profiles (central slices) of all traced atoms from the raw tomogram sliced perpendicular to the *x*-direction (**a**), the *y*-direction (**b**), and the *z*-direction (**c**). **d**-**f** Averaged intensity profiles (central slices) of all traced atoms from the DL augmented tomogram sliced perpendicular to the *x*-direction (**d**), the *y*-direction (**e**), and the *z*-direction (**f**). The pixel size is 0.357 Å.

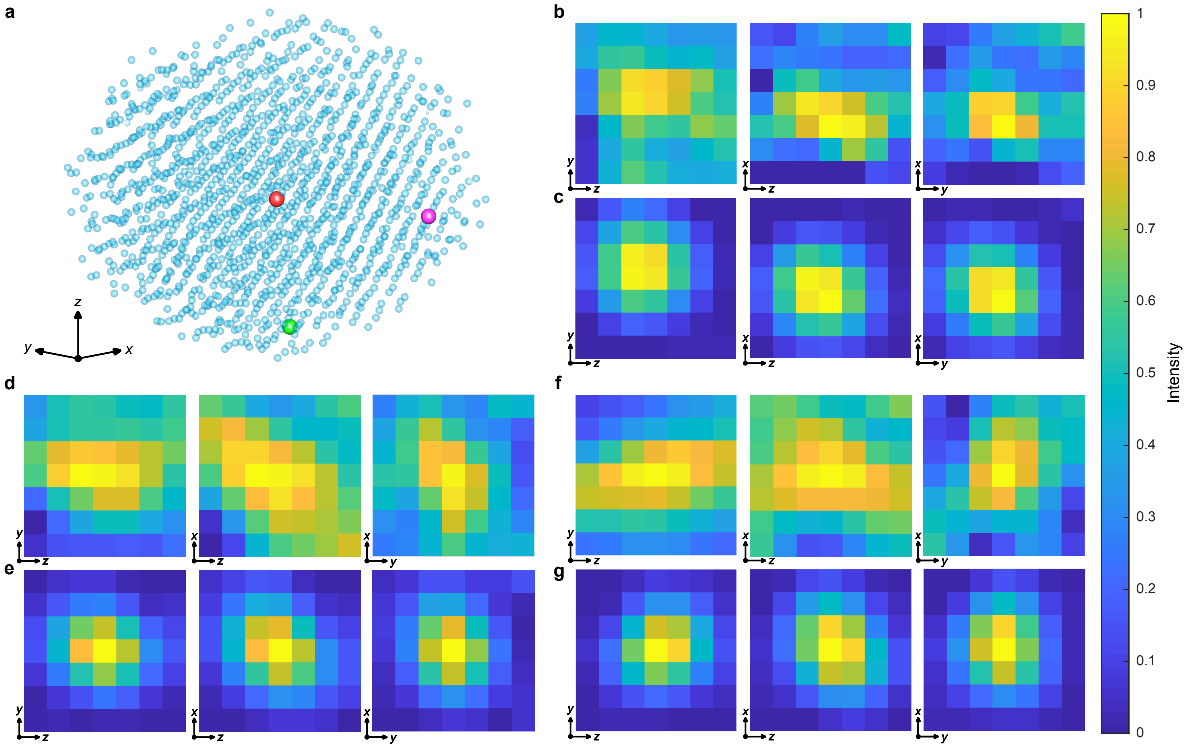

Supplementary Figure 16 | Representative 3D atomic profiles obtained from the experimental tomogram before and after the DL augmentation. a The atomic structure of the Pt nanoparticle after the DL augmentation. **b-c** Intensity profile (central slices) of an atom near the surface along the direction perpendicular to the missing wedge direction [the purple atom in (**a**)] before (**b**) and after (**c**) the DL augmentation. **d-e** Intensity profile (central slices) of an atom near the surface along the missing wedge direction [the green atom in (**a**)] before (**d**) and after (**e**) the DL augmentation. **f-g** Intensity profile (central slices) of an atom near the core of the nanoparticle [the red atom in (**a**)] before (**f**) and after (**g**) the DL augmentation. The pixel size is 0.357 Å.

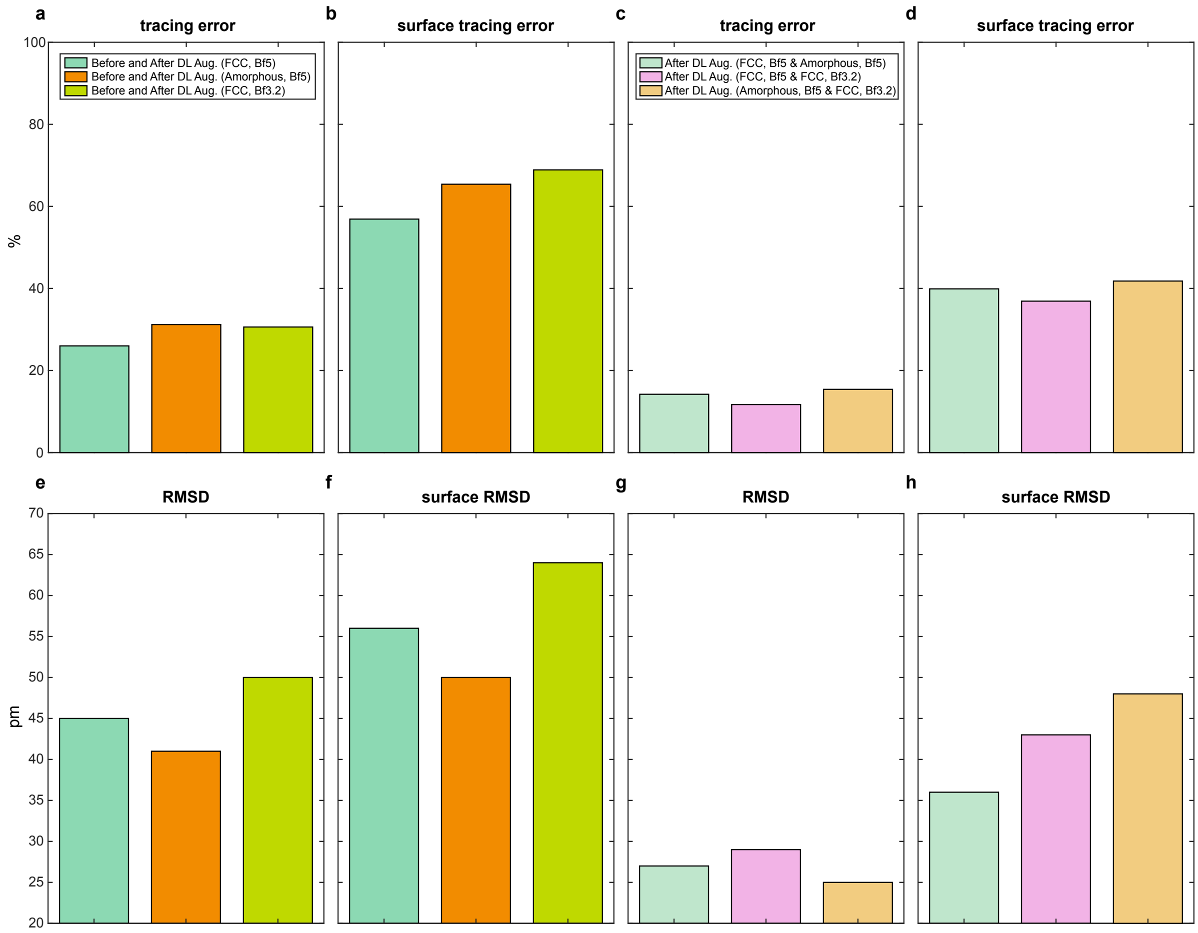

Supplementary Figure 17 | Tracing errors and RMSDs between the atomic structures obtained from the experimental data before and after different DL augmentations. The tracing errors (**a**-**d**) and RMSDs (**e**-**h**) were calculated from the obtained atomic structures i) by comparing the result from the raw tomogram to those from the DL augmented tomograms (**a**, **b**, **e**, **f**), and ii) by comparing between the results from the three different DL augmentations (**c**, **d**, **g**, **h**). (**a**, **c**, **e**, **g**) show the total tracing errors and RMSDs, and (**b**, **d**, **f**, **h**) show the surface tracing errors and RMSDs.

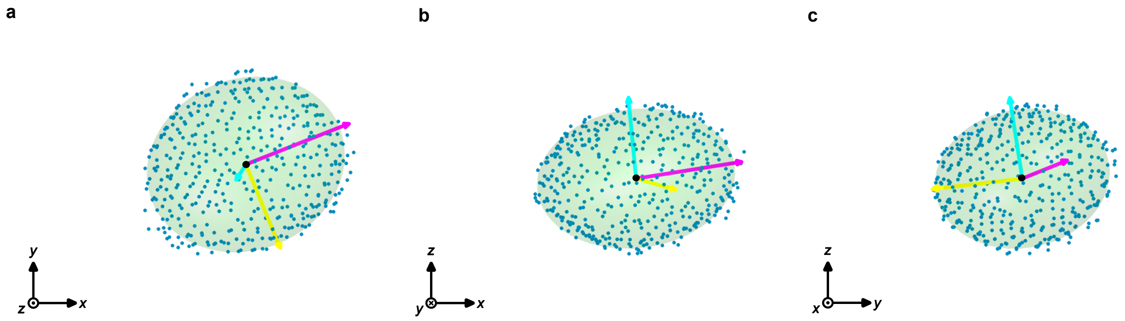

Supplementary Figure 18 | Fitted ellipsoid to surface atoms of the Pt nanoparticle. a-c An ellipsoid was fitted to the surface Pt atom positions to identify the overall shape and orientation of the nanoparticle. Blue dots represent the surface Pt atoms. The axes colored with three different colors represent the direction of principal semi-axes of the fitted ellipsoid. The magenta, cyan, and yellow axis directions are [0.92, 0.36, 0.15], [0.38, -0.92, -0.11], and [-0.10, -0.15, 0.98] in the lab-coordinates, respectively.

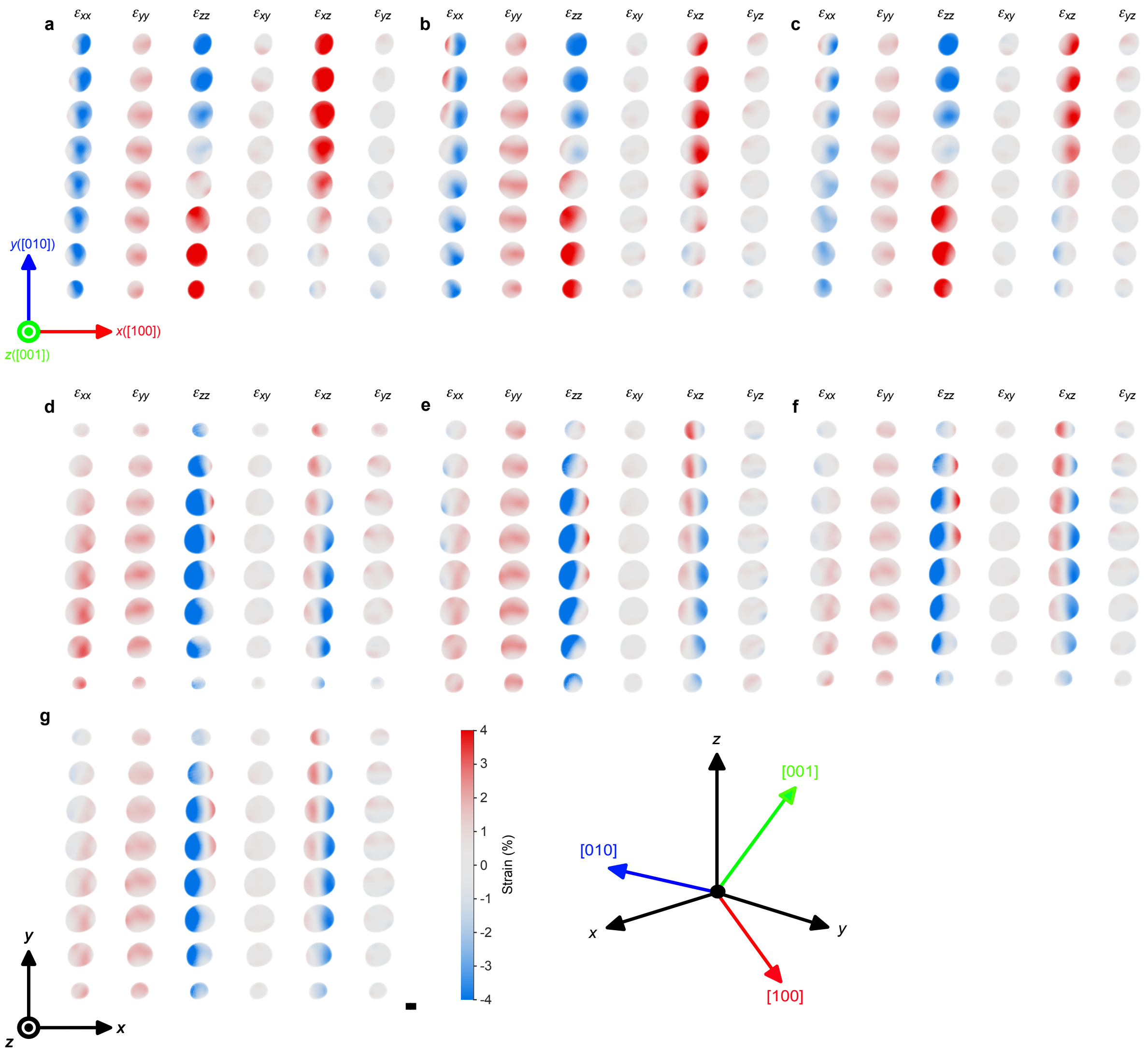

Supplementary Figure 19 | 3D Strain maps calculated from different axis conventions and different DL augmentations. The strain tensors calculated based on the f.c.c. crystallographic axis convention (**a**-**c**) and the lab-coordinate axis convention (**d**-**g**) by traced atoms from the raw tomogram (**a**, **d**), the tomograms after the DL augmentation trained by f.c.c. models + Bfactor 5 Å^2 (**g**), amorphous models + Bfactor 5 Å^2 (**b**, **e**), and f.c.c. models + Bfactor 3.2 Å^2 (**c**, **f**). Scale bar, 2 nm.

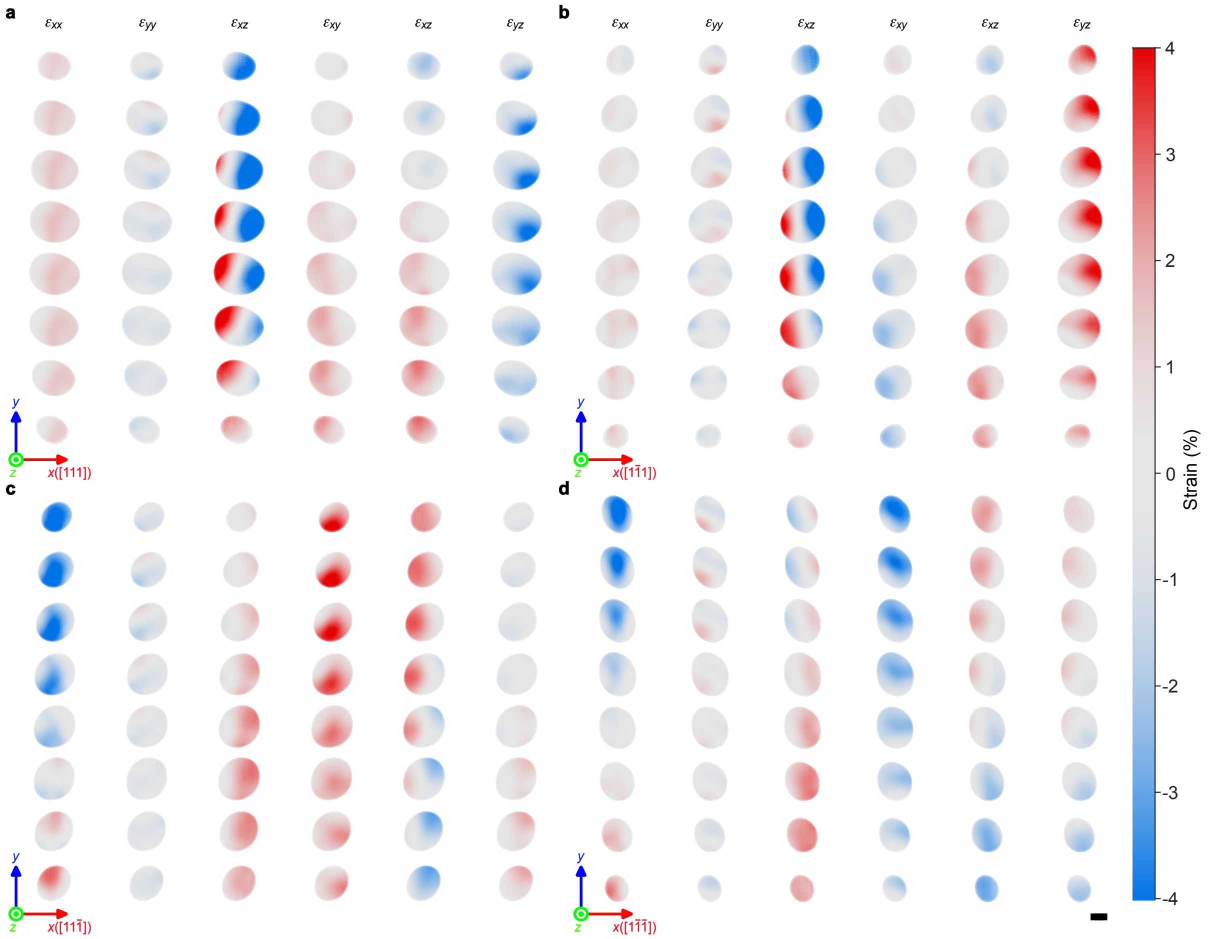

Supplementary Figure 20 | 3D strain maps calculated from <111> axis conventions. The strain tensors calculated for four different axes conventions with respect to the f.c.c. crystallographic axes. The x-directions are (**a**) [111], (**b**) $[1\bar{1}1]$, (**c**) $[11\bar{1}]$, (**d**) $[1\bar{1}\bar{1}]$ directions, respectively. Clear tensile strain is observed along the [111] and $[1\bar{1}1]$ directions (ε_{xx} maps of (**a**) and (**b**)) which are at the opposite side of the particle-substrate interface. On the other hand, along the $[11\bar{1}]$ and $[1\bar{1}\bar{1}]$ directions (which are toward the substrate), compressive strains can be observed for the ε_{xx} maps. All strain maps were calculated from the atomic structure obtained from the experimental data after the DL augmentation trained by f.c.c.-based atomic model with Bfactor 5 Å^2. Scale bar, 2 nm.

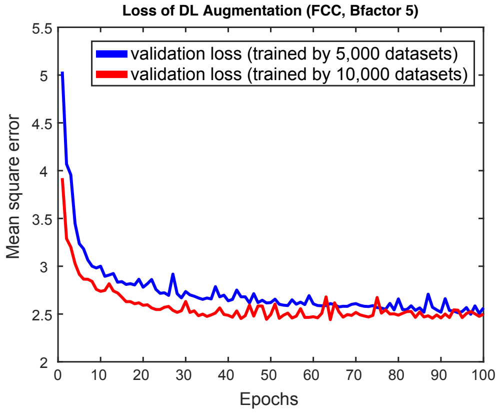

Supplementary Figure 21 | Learning curves of the DL augmentation networks trained by different number of training datasets. The learning curves represent the validation losses as a function of training epochs. The blue and red lines show the validation losses during the trainings with 5,000 and 10,000 tomograms, respectively.

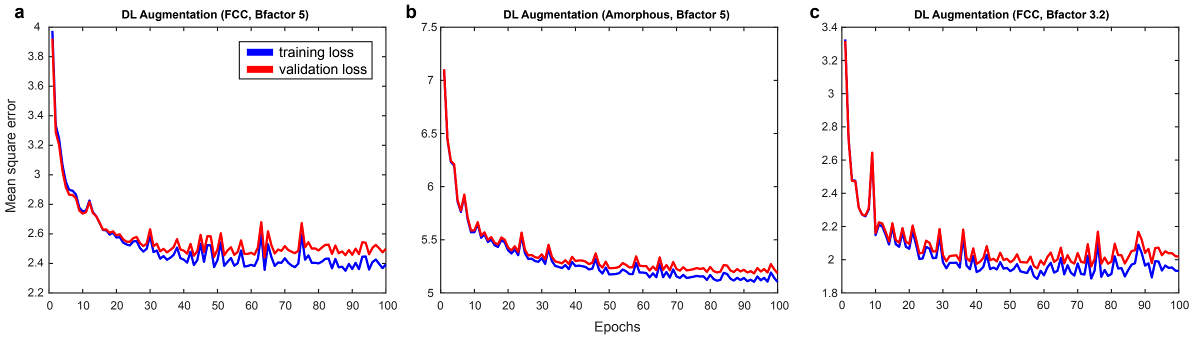

Supplementary Figure 22 | Learning curves of the DL augmentation networks for three different training datasets. The learning curves during the training of the networks based on (**a**) the f.c.c. atomic models with Bfactor 5 Å^2, (**b**) the amorphous atomic models with Bfactor 5 Å^2, and (**c**) the f.c.c. atomic models with Bfactor 3.2 Å^2. The blue and red solid lines represent the losses from training datasets and validation datasets, respectively.

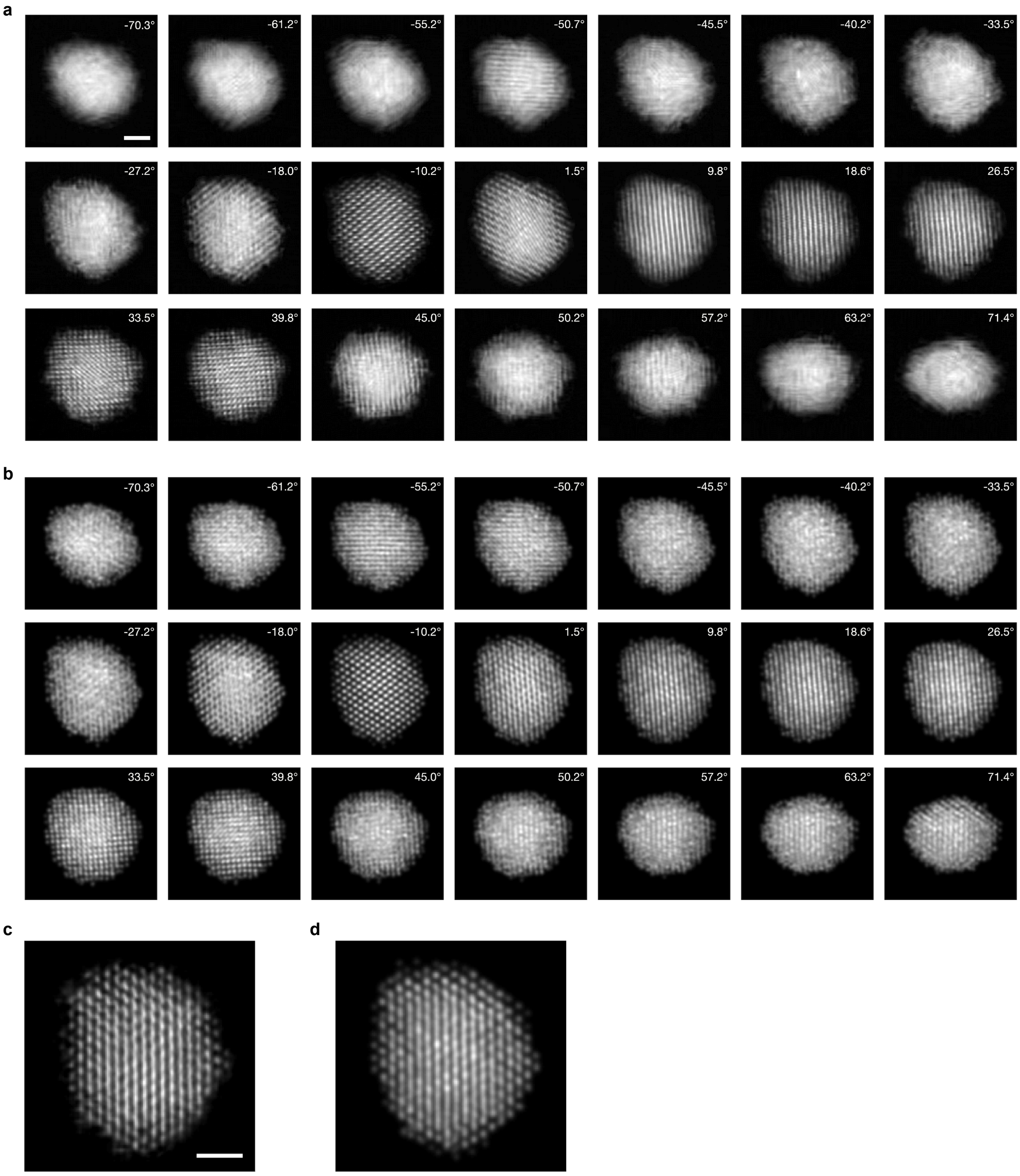

Supplementary Figure 23 | Experimental tilt series and forward-projected tilt series from the final atomic model. a. The post-processed tilt series images acquired from the ADF-STEM experiment (Methods). The angles denoted at the top right corner of each image represent the refined θ angles after the angle refinement procedure (Methods). **b.** Forward-projected tilt series from final 3D atomic models along the refined final tilt angles. Electron scattering factors of Pt atoms and Gaussian broadening (Bfactor 5 Å^2) were considered for the forward projections. Averaged R-factor between the tilt series images in (**a**) and (**b**) is 0.173. **c**. The experimental zero-degree projection acquired right after the tilt series acquisition. **d**. The forward projection of the final 3D atomic model along the angle which gives the best consistency with the post-experiment zero-degree projection (**c**). The best consistency angle was determined to be ψ : 4°, θ : −3.5°, φ: 1°. The excellent consistency between **c** and **d** demonstrates that the surface and internal atomic structure of the nanoparticle did not change by the tilt series acquisition, and the final 3D atomic model is reliable. Scale bars, 1 nm.
